# Emergence of *Orientia tsutsugamushi* as an important cause of Acute Encephalitis Syndrome in India

**DOI:** 10.1371/journal.pntd.0006346

**Published:** 2018-03-28

**Authors:** Parul Jain, Shantanu Prakash, Piyush K. Tripathi, Archana Chauhan, Shikha Gupta, Umesh Sharma, Anil K. Jaiswal, Devraj Sharma, Amita Jain

**Affiliations:** 1 Department of Microbiology, King George’s Medical University, Lucknow, India; 2 Department of Microbiology, Patna Medical College, Patna, Bihar, India; 3 Department of Pediatrics, Patna Medical College, Patna, Bihar, India; University of North Carolina at Chapel Hill, UNITED STATES

## Abstract

**Background:**

Acute Encephalitis Syndrome (AES) is a major seasonal public health problem in Bihar, India. Despite efforts of the Bihar health department and the Government of India, burden and mortality of AES cases have not decreased, and definitive etiologies for the illness have yet to be identified.

**Objectives:**

The present study was undertaken to study the specific etiology of AES in Bihar.

**Methods:**

Cerebrospinal fluid and/or serum samples from AES patients were collected and tested for various pathogens, including viruses and bacteria by ELISA and/or Real Time PCR.

**Findings:**

Of 540 enrolled patients, 33.3% (180) tested positive for at least one pathogen of which 23.3% were co-positive for more than one pathogen. Most samples were positive for scrub typhus IgM or PCR (25%), followed by IgM positivity for JEV (8.1%), WNV (6.8%), DV (6.1%), and ChikV (4.5%).*M*. *tuberculosis* and *S*. *pneumoniae* each was detected in ~ 1% cases. *H*. *influenzae*, adenovirus, Herpes Simplex Virus -1, enterovirus, and measles virus, each was detected occasionally. The presence of Scrub typhus was confirmed by PCR and sequencing. Bihar strains resembled Gilliam-like strains from Thailand, Combodia and Vietnam.

**Conclusion:**

The highlights of this pilot AES study were detection of an infectious etiology in one third of the AES cases, multiple etiologies, and emergence of *O*. *tsutsugamushi* infection as an important causative agent of AES in India.

## Author summary

Acute encephalitis syndrome (AES) is a dreaded disease in India including the state of Bihar. Every year several people specially children, succumb to this disease and often the survivors are left with permanent residual disorders. The present research throws light on specific etiological agents that may cause AES and have found scrub typhus to be an important etiology. Knowing the specific etiology would help in definitive management of the patients that may improve the outcome both in terms of morbidity and mortality, as well as help the policy makers to take specific action for prevention and control of the disease.

## Introduction

Acute Encephalitis Syndrome (AES) is a major seasonal public health problem in many states of India including Bihar. Muzaffarpur and adjacent districts, including Sitamarhi, Sheohar and East Champaran districts of Bihar consistently experience a great burden of the disease [1].The mean numbers of reported AES cases and deaths per year from Bihar during 2011 to 2014 were 835 and 243 respectively. During these years, the state of Bihar contributed 9.5% and 18% of the mean number of AES cases and deaths respectively reported from all over India (data from NVBDCP website) [2]. Japanese Encephalitis virus (JEV) was hitherto considered as the most important cause of AES, however, it contributed to only 20 (1.5%) of AES cases in 2014[2]. Other etiologies, including enteroviruses and Nipah Virus have also been implicated [1]. The National Centre for Disease Control (NCDC), New Delhi and the Global Disease Detection, Regional Centre, India, Center for Disease Control and prevention (CDC), US, have jointly classified Muzaffarpur, Bihar mystery to a noninfectious toxic encephalopathy associated with consumption of litchi fruit after ruling out pesticides, heavy metal poisoning and infectious diseases [3]. However, despite the efforts of the Bihar health department and the Government of India, the burden and mortality of AES cases have not decreased, and definitive etiologies for these illnesses have yet to be identified.

Identification of a specific agent is important for patient management and for understanding the epidemiology. Therefore, the present study was undertaken to study the specific etiology of AES in Bihar, India.

## Materials and methods

### Study sites

The patient enrollment and sample collection center was Patna Medical College and Hospital (PMCH) Patna (Coordinates: 25.5941° N, 85.1376° E), a tertiary care referral center catering to the population of Patna and the surrounding districts. The testing centers were (1) the Virus Research and Diagnostic Laboratory (VRDL) at King George’s Medical University (KGMU), Lucknow, Uttar Pradesh, and (2) VRDL at PMCH, Patna.

### Study population and samples collected

Cases presenting with clinical diagnosis of AES as per WHO [4] and admitted at PMCH were enrolled in the study during June 2015 to September 2016. Depending on the feasibility, samples of serum, CSF or both were collected from each case after obtaining written informed consent from the patient. In case of unconscious patient or children written informed consent was obtained from guardian of the patient. The study was approved by the institutional ethics committee (Reference code: 83^rd^ ECM IIA/P8).

### Laboratory tests

**Fig 1:**
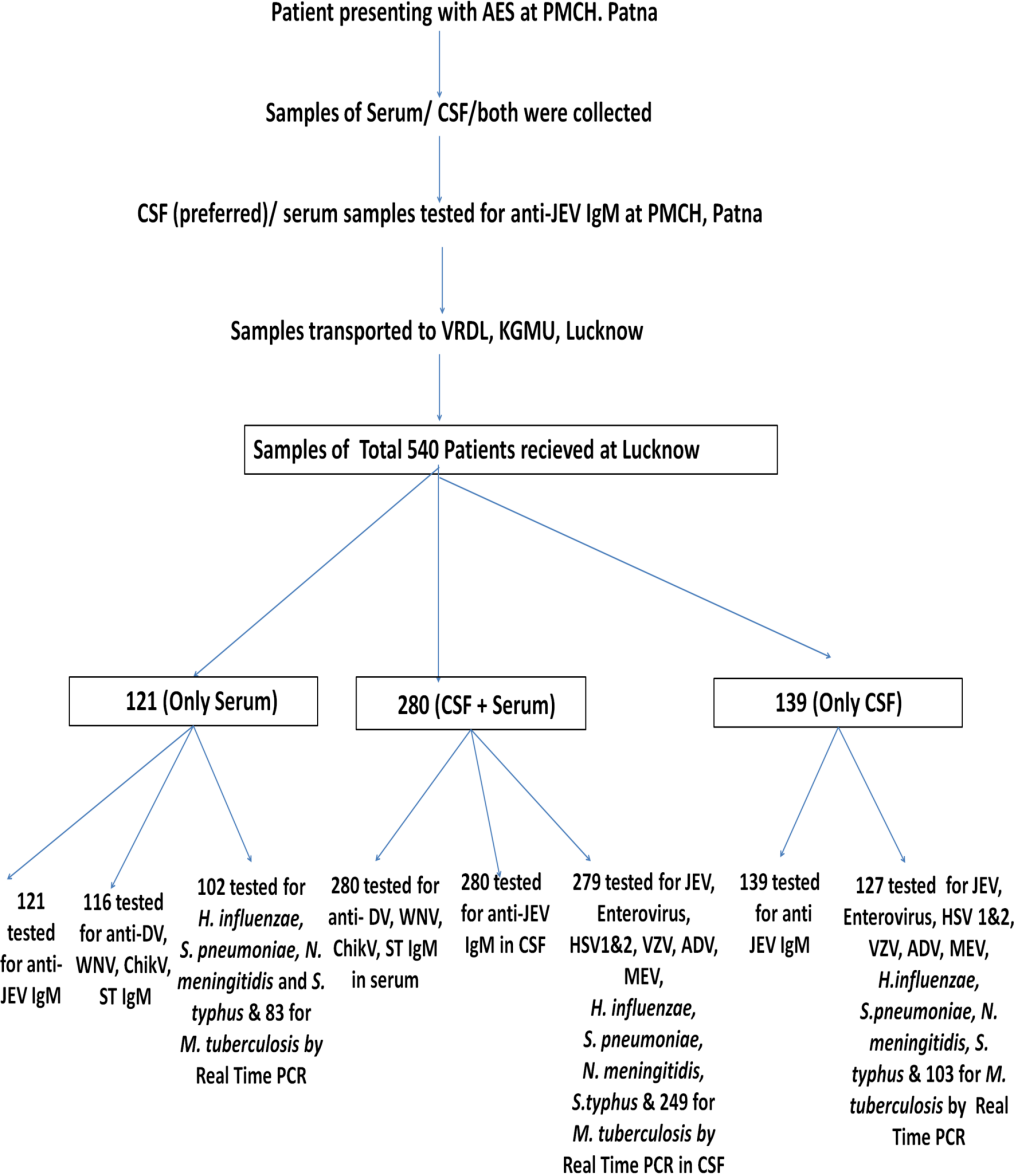
Patient enrolment, samples collected and tests done in patients enrolled in the study.

At the VRDL of PMCH, Patna, the CSF (preferred)/ serum (in the absence of CSF) sample was tested the same day for anti- JEV IgM antibodies (MAC ELISA kits manufactured by the National Institute of Virology, Pune, India) and the remaining samples were transported to the VRDL at KGMU Lucknow in dry ice for further testing (Fig 1).

At KGMU, the serum samples were tested by ELISA for anti-Dengue Virus (DV) IgM and anti- Chikungunya Virus (Chik V) IgM, using kits by the National Institute of Virology, Pune, India. Anti-West Nile Virus (WNV) IgM, and anti-Scrub typhus IgM (ST) antibodies were tested using Inbios International, USA kits. For anti-Scrub typhus IgM, samples with an optical density (OD) >0.5 were considered positive. For scrub typhus IgM the baseline titres need to be established for each region; for India this value has been calculated as 0.5 [5]. For all the other ELISAs the cut off values were calculated based upon the manufacturer’s instructions. All ELISAs were done in serum samples except anti-JEV IgM ELISA, which was preferably done in CSF samples (as per the manufacturer’s recommendations). External Quality Assessment for PMCH, Patna anti-JEV IgM ELISAwas done atVRDL, KGMU. Total 40% and 15% of the anti- JEV IgM antibody positive and negative samples respectively, were retested by ELISA using the same kit and protocol. All results from both the laboratories were concordant.

All Real time PCRs were done in CSF samples, except bacterial PCRs which were done in serum samples in case CSF was not available (Fig 1).

### Nucleic acid extraction and Real Time PCR

RNA was extracted from 140 μl processed clinical samples using the QIAamp Viral RNA mini kit (Qiagen, Hilden, Germany) following the manufacturer’s protocol. For DNA extraction, QIAamp Viral DNA mini kit (Qiagen, Hilden, Germany) was used. All the PCRs were done using Taqman chemistry. The target gene used for selecting the primer and probes of various pathogens, their product size and their references are described in Table 1. For Enterovirus, JEV and VZV the primers and probe were self-designed (Table 1). The properties of the primers were analyzed by IDT oligoanalyzer software. Amplification was done with Real Time PCR machine (ABI 7500, Applied Biosystems, USA). The Ct value of 35 was taken as the cutoff.

**Table 1:** Details of primers and probe used for real time PCR.

Virus	Target Gene	Amplicon size (base pairs)	Reference
Enterovirus*	5’ NTR	144bp	*Self Designed* EnteroV Fwd: CCTGAATGCGGCTAATCCYAA EnteroV Rvs: TTGTCACCATAAGCAGCCA EnteroV Probe: CCGACTACTTTGGGTGTCCGTGTT (5FAM/3IABkFQ)
Adenovirus^#^	hexon region	96bp	[29]
JEV*	Core gene	116bp	*Self Designed* JEV Fwd: ATGGATCACRGAMTATGCGGG JEV Rvs: TGCGRTTGAGYTGGATRAC JEV Probe: TYGCAATGTGCCTYCAAAGAGCG (5HEX/3IABkFQ)
HSV-1^#^	UL36	143-151bp	Patent WO2016071925 A3 (Jain *et al*., 2016)
HSV-2^#^	UL27	141bp	Patent WO2016071925 A3 (Jain *et al*., 2016)
VZV^#^	ORF 29	171bp	Self Designed VZV Fwd: CAAAGGCAACCATGCAGGACACTT VZV Rvs: TACGCGCACGCAGTGTGTCTAATA VZV Probe: TGTGGATCATCCAACGTTTCGTCGCA (5HEX/3IABkFQ/)
Measles virus*	nucleoprotein (N) gene	114bp	[30]
*H*. *influenzae*^#^	protein D (Hpd)	113bp	Patent WO2012103353A2 (Jennifer Dolan Thomas, 2012)
*S*. *pneumoniae*^#^	Autolysin (lyt A)	75bp	[31]
*N*. *meningitidis*^#^	capsule transport to cell surface gene (CtrA)	114bp	[31]http://www.cdc.gov/meningitis/lab-manual/chpt10-pcr.html
Scrub typhus^#^	gene for a 47-kDa protein (Htr A)	118bp	[32]
*M*. *tuberculosis*^#^	IS6110 gene	163bp	[33]

*^#^For Real Time PCR: Final Volume: 25μl, Nucleic acid: 7.5μl, Each Primer (10pmol): 0.5μl, Probe (5pmol): 0.5 μl

* For real time PCR of RNA pathogens

Reaction mixture: 12.5μl of 2x RT-Buffer + 1μl of 25x RT enzyme (AgPath, Life technologies, CA, USA).

Amplification conditions for RNA pathogens: Pre amplification at 45°C for 10 minutes, 1 cycle at 95°C for 10 minutes, 45 cycles of 95°C for 15 sec, 60°C for 1 minute

^#^For real time PCR of DNA pathogens

Reaction mixture: 12.5μl TaqMan Universal PCR Master Mix (Life technologies, CA, USA).

Amplification conditions for DNA pathogens: 1 cycle at 95°C for 10 minutes, 45 cycles of 95°C for 15 sec, 60°C for 1 minute

### Sequencing

For samples testing positive for scrub typhus DNA, sequencing was done for the 56 kDa TSA gene region with a nested PCR [6] using a high fidelity Taq polymerase (Thermo Fisher Scientific, Waltham, MA). The gene sequence thus obtained spanned three of the four major variable regions. Sequencing was performed by utilizing two sets of primers as described by Ruang-areerate T. The outer primers were JG-OtF584 (5’-CAA TGT CTG CGT TGT CGT TGC) and RTS9 (5’-ACAGAT GCA CTA TTA GGC AA), and the inner primers were F (5’-AGC GCTAGG TTT ATT AGC AT) and RTS8 (5’-AGG ATT AGA GTG TGG TCCTT) [6].The PCR product was sequenced bidirectionally using the Big Dye Terminator cycle sequencing kit (Applied Biosystems, Foster City, CA) and ABI Prism Genetic Analyzer 3130 (Applied Biosystems). The GenBank accession numbers obtained for the sequences from this study are MG940993 to MG940998.

### Phylogenetic analysis

Phylogenetic analysis was performed and a tree was constructed using the Maximum Likelihood Method (Tamura-Nei model) and the MEGA version 6 program. The number of bootstrap replications was set to default. Phylogenetic tree was constructed using the sequences obtained and the reference sequences retrieved from the GenBank database (Karp, AY956315.1; Kato, AY836148.1; Gilliam, HQ718429.1 (Cambodia), HQ718460.1 (Vietnam), EF213099.1 (Thailand) and DQ485289.1).

### Ethics statement

The study was approved by the institutional (King George’s Medical University) ethics committee. Samples were collected after obtaining written informed consent from the patient or guardian in unconscious patients/ children.

### Statistical analysis

All statistical analyses were done using GraphPad Prism software version 5. Intergroup comparisons of categorical and continuous variables were done using Fischer’s exact test and Chi square tests respectively.

## Results

Total 540 patients were enrolled. Both serum and CSF samples were obtained from 280 cases and only CSF and only serum were obtained from 139 and 121 cases respectively. Due to limited availability and quantity of sample, all the tests could not be performed in all the cases. The testing details are given in Fig 1.

Total 521/540 (96.5%) cases were children (aged< 180 months old, Mean age: 84.4 months, Range: 2 months to 78 years) and 312 (57.8%) were males (Male to female ratio; 1.4:1). Total 33.3% (180 of 540) patients tested positive for at least one pathogen. The total positivity of all the etiological agents combined together was not significantly different between age or sex groups (Table 2).

**Table 2:** Test positivity and age and sex distribution of total cases.

Age Group (In months)	Male (n = 312)		Female (n = 228)		Total (n = 540)		P value (Chi^2^)
	Tested	Positive (%)	Tested	Positive (%)	Tested	Positive (%)	
0–60	153	50 (32.7)	102	27 (26.5)	255	77 (30.2)	0.35 (3.2)
61–120	106	37 (34.9)	89	37 (41.6)	195	74 (37.9)	
121–180	40	11 (27.5)	31	11 (35.5)	71	22 (30.9)	
>180	13	4 (30.7)	6	3 (50)	19	7 (36.8)	
Total	312	102 (32.7)	228	78 (34.2)	540	180 (33.3)	
Mean Age (in months) ±SD	85.8±88.93		82.4±66.01		84.4±80.32		
**P Value (95%CI)**	0.71 (0.75–1.2)						

Most samples were positive for scrub typhus IgM or PCR (25%), followed by IgM positivity for JEV (8.1%), WNV (6.8%), DV (6.1%), and ChikV (4.5%). (Table 3). Since many samples were co-positive for 2 or more antibodies, the exact proportion of each agent could not be known. *M*. *tuberculosis and S*. *pneumoniae* each was detected in approximately 1% cases. *H*. *influenzae*, adenovirus, HSV-1, enterovirus, and measles virus, each were detected in less than 1% cases. *N*. *meningitidis*, HSV-2 and VZV were not detected in any case (Table 3).

**Table 3:** Laboratory results of AES cases from Bihar.

Sample	Test Name	Total Tested	Positive (%)	Co-positives (% of positives)
CSF (preferred)/ Serum	Anti JEV IgM	540	44 (8.1)	22 (50)
	*H*. *influenzae*	508	3 (0.6)	0 (0)
	*S*. *pneumoniae*	508	5 (1.0)	0 (0)
	*N*. *meningitidis*	508	0 (0)	0 (0)
	*M*. *tuberculosis*	435	4 (0.9)	0 (0)
Serum	Anti DV IgM	396	24 (6.1)	20 (83.3)
	Anti ChikV IgM	396	18 (4.5)	13 (72.2)
	Anti WNV IgM	396	27 (6.8)	17 (62.9)
	Anti scrub typhus IgM	396	99 (25)	18 (18.2)
CSF	Enterovirus Real Time PCR	399	1 (0.3)	1 (100)
	Adenovirus Real Time PCR	399	3 (0.8)	1 (33.3)
	HSV-1 Real Time PCR	399	2 (0.5)	1(50)
	HSV-2 Real Time PCR	399	0 (0)	0 (0)
	VZV Real Time PCR	399	0 (0)	0 (0)
	Measles virus Real Time PCR	399	2 (0.5)	1 (50)
	JEV Real Time PCR	300	0 (0)	0 (0)
Total			232*	

*Some samples were co-positive for >two pathogens. Therefore, etiology could be determined in only 180 cases.

Of the cases testing positive, co-detection of more than one pathogen was seen in 23.3% (42/180) cases; the co-detection of antibodies against more than one arboviruses was more common. The frequency varied from 50% for anti JEV IgM to 83.3% for anti- DV IgM (p value = 0.04, Chi square = 8.14). Difference between co-detection among flaviviruses (JEV, DV, WNV) and alphavirus (ChikV) was not statistically significant (p value = 0.59, 95% CI: 0.79–1.09). Anti- scrub typhus IgM antibodies showed a significantly lower co-detection than the arboviruses antibodies (p value<0.0001, 95% CI: 1.93–3.32) (Table 3). The different combinations of co-positives are shown in Table 4. Simultaneous detection of nucleic acid of more than one pathogen was found in only one case (scrub typhus and HSV1 DNA).

**Table 4:** Co detection of more than one pathogen: Combinations detected.

Combinations	Number
**Co-positivity between Arboviruses**	
Anti ChikV IgM+ Anti DV IgM+ Anti JEV IgM	2
Anti ChikV IgM+Anti DV IgM	3
Anti ChikV IgM+Anti JEV IgM	4
Anti DV IgM+ Anti JEV IgM + Anti WNV IgM	3
Anti JEV IgM + Anti WNV IgM	7
Anti JEV IgM + Anti DV IgM	2
Anti DV IgM+ Anti WNV IgM	3
**Co-positivity between scrub typhus and viruses**	
Scrub typhus PCR + HSV1 PCR	1
Enterovirus PCR + Anti scrub typhus IgM	1
Anti ChikV IgM+Anti scrub typhus IgM+Anti DV IgM	2
Anti ChikV IgM+Anti scrub typhus IgM	2
Anti scrub typhus IgM+ Anti JEV IgM+ Anti WNV IgM	2
Anti scrub typhus IgM+ Anti WNV IgM	2
Anti scrub typhus IgM+ Anti JEV IgM	1
Anti scrub typhus IgM+ Anti DV IgM	5
Anti scrub typhus IgM+ Anti JEV IgM+ Measles virus RNA	1
Adenovirus PCR + Anti scrub typhus IgM	1
Total number of cases showing co- detection	42

The clinical features were available for 124 patients out of 180 patients positive for any pathogen. The most common clinical features were fever (100%, n = 124), altered sensorium (79.8%, n = 99), headache (71.8%, n = 89), nausea/ vomiting (n = 53.2%, n = 66), seizures (50.8%, n = 63), and neck rigidity (32.3%, n = 40). No significant difference in clinical features was seen in cases with different etiologies.

**Fig 2:**
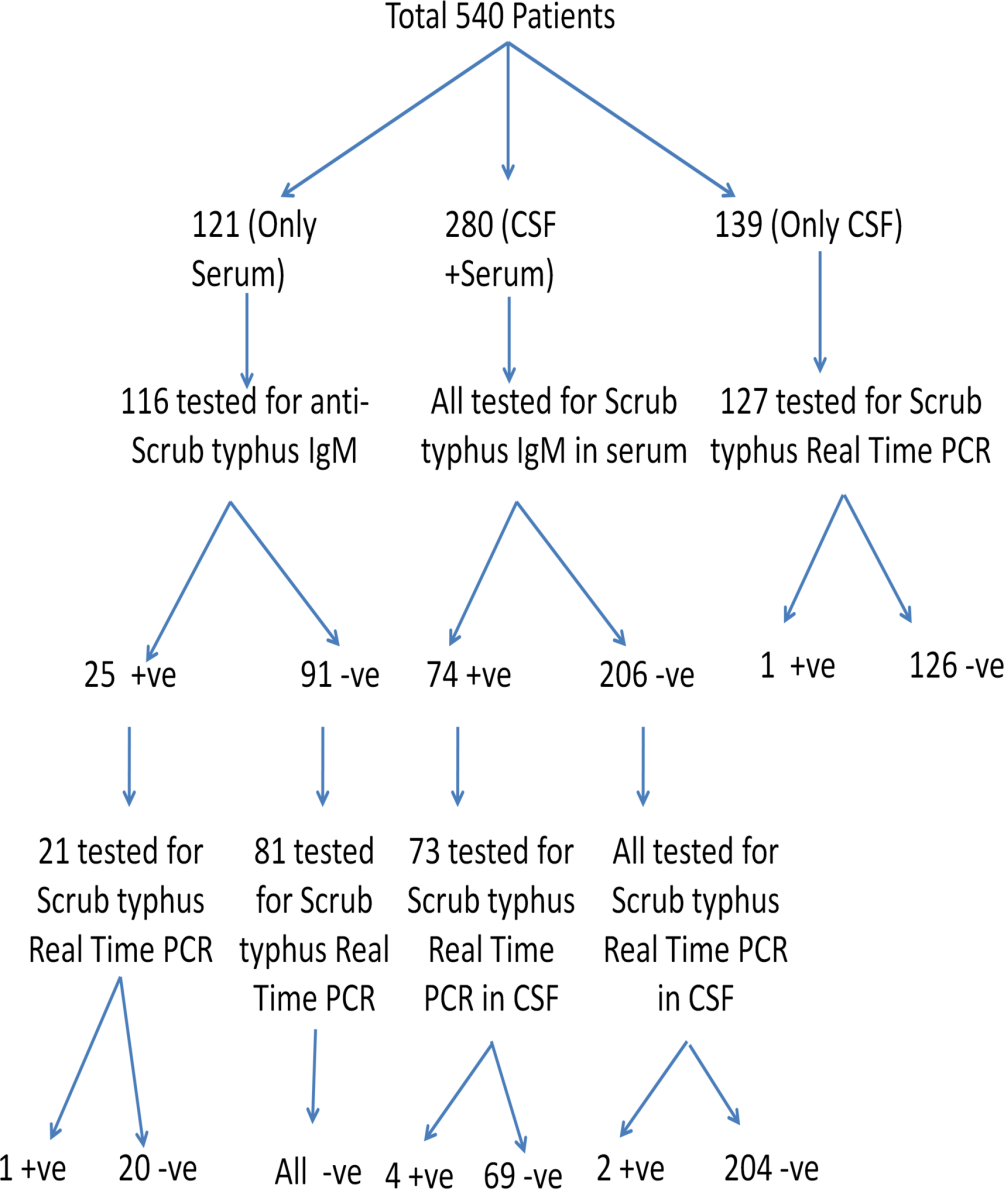
Results of scrub typhus in AES cases from Bihar.

**Fig 3:**
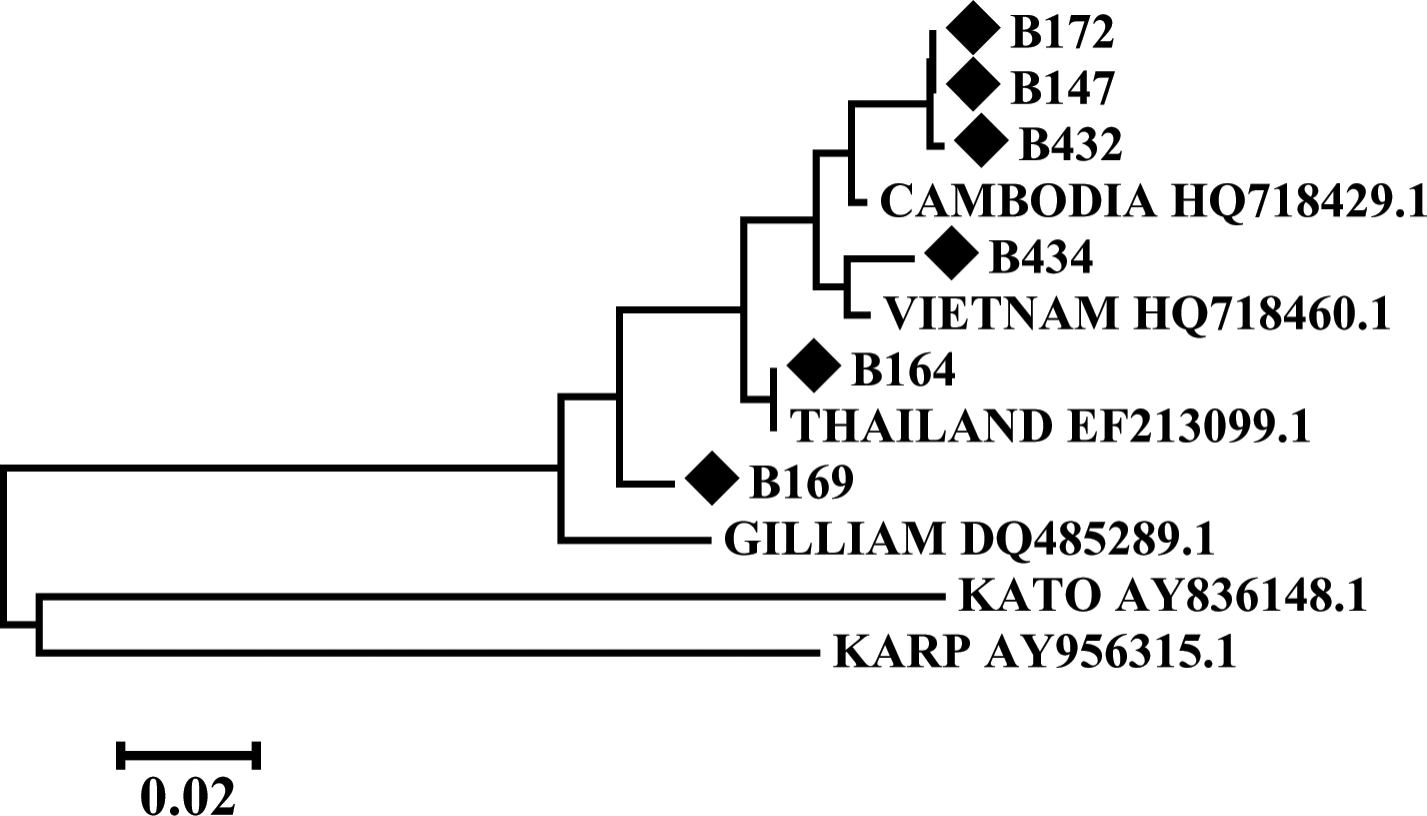
Phylogenetic analysis of scrub typhus strains by Maximum Likelihood Method using MEGA6. The tree is drawn to scale, with branch lengths measured in the number of substitutions per site. There were a total of 602 positions in the final dataset. The six sequences obtained (shown in bullets) were compared to Gilliam, Karp and Kato prototype strains and to the closest matching sequences obtained by BLAST analysis.

Since scrub typhus was the most common etiology detected and AES due to scrub typhus has not been reported from Bihar till date, scrub typhus real time PCR was done in cases where samples were available. CSF was preferred over the serum sample (Fig 2). The PCR detected total eight cases of scrub typhus of which five also had anti-scrub typhus IgM antibodies. Six of eight Real Time PCR positive samples could be sequenced, which on BLAST analysis showed a maximum similarity with the Thailand, Cambodia and Vietnam scrub typhus strains. On conducting a molecular phylogenetic analysis by Maximum Likelihood method based on the Tamura-Nei model in MEGA6 software, the scrub typhus sequences obtained clustered with Gilliam like strains (Fig 3).

**Fig 4:**
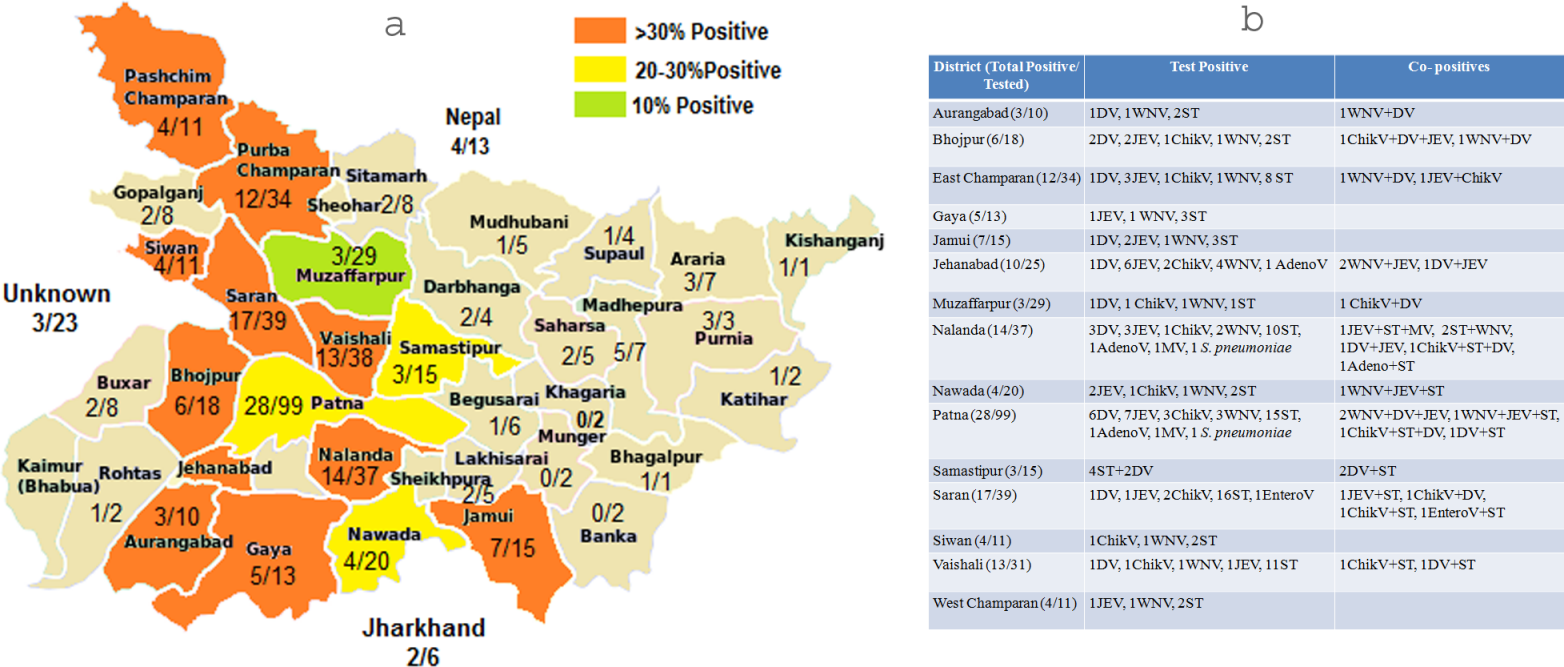
Geographic distribution of cases referred to the virology laboratory. 4a: Bihar district map showing number of cases positive for any test/ total cases referred from that district. 4b: Table showing district wise distribution of positives and co-positives in those referring more than 10 cases to the virology laboratory.

Most of the patients were referred from Patna and its surrounding districts. Nepal and Jharkhand (shared boundaries) referred 13 and 6 cases respectively. Geographic location of 23 patients could not be traced (missing data). Analysis was done only for 15 districts referring more than 10 cases, of which, eleven showed overall high positivity (>30% positives), three districts showed moderate positivity (>20–30%) and one (Muzaffarpur) showed low positivity (10%) (p value = 0.0014, Chi square = 13.14) (Fig 4A). District wise total number of samples referred, total number of tests positive, the names of tests positive and the co-positives are mentioned in Fig 4B.

**Fig 5:**
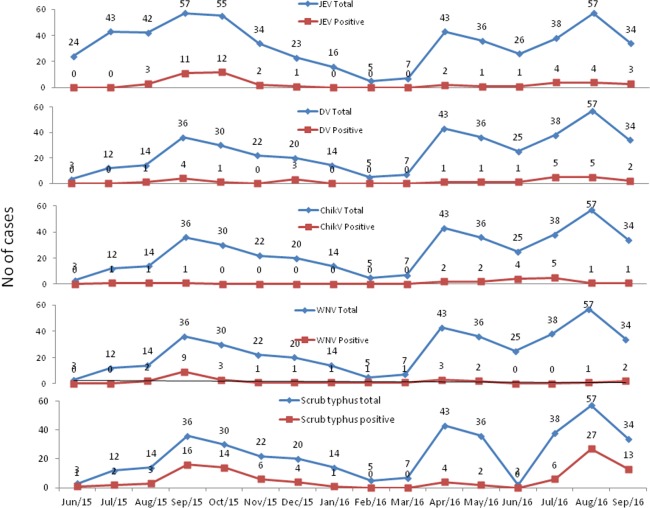
Seasonal distribution of total AES cases referred and arbovirus and scrub typhus positivity.

A month wise analysis was done on the total AES cases referred to the virology laboratory and of arbovirus positivity (Fig 5). AES cases were reported throughout the year with a dip in the number of cases during February and March. Similarly, anti-DV IgM and anti-WNV IgM were positive throughout the year, but with a small peak during August through October. Anti-JEV IgM and anti ChikV IgM showed a distinct seasonality with maximum number of cases being observed during August to October and during June through July respectively. Scrub typhus peaks were also seen during September and October.

## Discussion

For over 20 years, the state of Bihar has witnessed periodic outbreaks of Acute Encephalitis Syndrome. The victims are usually malnourished children, with the median age been reported from 4–5 years. In the present study most of the AES cases were children less than 15 years of age. The disease has no remarkable sex preference as was also observed over the years during the Bihar AES outbreaks from 2011 to 2014 [7].

We screened all the patients for DV, WNV and ChikV because all these flaviviruses are closely related to JEV and are known to cause AES. We screened for scrub typhus since the organism *Orientia tsutsugamushi* has increasingly been recognised as a cause of AES [8,9]. Enteroviruses, HSV, VZV, and Adenovirus are already established causes of AES. We also wanted to know the percentage of bacterial infections presenting as AES as these are easily treatable conditions, provided patient is diagnosed and treated in a timely manner.

An infectious etiology could be determined in about one-third of the AES cases, which was a mixed pot showing the simultaneous presence of several pathogens. This observation is similar to that obtained from AES cases from Uttar Pradesh [10]. Since few samples were referred from each district, we could not draw any conclusion from the geographical distribution of the cases. JEV, ChikV and scrub typhus showed a definite seasonality with an increase in the number of cases in the monsoon and the post monsoon season as per the previous studies [11,12]. Seasonality of DV cannot be commented upon as most of the anti DV IgM positives were co-positives with antibodies against other pathogens.

JEV was found only in about 8% cases. In the year 2012–13 the Government of India initiated JE vaccination and other AES/JE control activities in following districts of Bihar i.e. Araria, East Champaran, West Champaran, Darbhangha, Gaya, Muzaffarpur, Gopalganj, Jehanabad, Nawada, Nalanda, Patna, Samastipur, Vaishali, and Saran [7]. We could not know the vaccination coverage in these districts. However, in the neighbouring state of Uttar Pradesh the JE vaccination coverage in 7 districts of the Lucknow region viz. Raebareli, Hardoi, Sitapur, Unnao, Lakhimpur Khiri, Lucknow ranged from 66.80% in the year 2014–15 to 76.54% in the year 2016–17 (personal communication with UP Vector Borne Disease Control Program). In north India, the protective efficacy of a single dose of SA-14-14-2 JE vaccine has been reported to be varying from 94.5% [13] to 84% [14]. JE vaccination program might have brought down the incidence of JE in Bihar.

Surprisingly, most of the data available from Bihar are only from Muzaffarpur district with limited data from other parts of the state. Several theories have been put forward by different researchers to explain the etiology of AES cases in Muzaffarpur district. These hypotheses include non-infectious, toxic encephalopathy due to the toxin methylenecyclopropyl-glycine present in lychee fruit, which causes hypoglycemia and encephalopathy on the background of malnourishment [3, 15]. Among infectious etiologies besides JEV, the usual cause of AES in India, Nipah virus was also thought as a possible etiology [1] because of a large number of bats being usually present in lychee orchards feeding on the lychee fruit, which were later consumed by the children. In 2014, the National Institute of Virology, Pune and the National Communicable Disease Center, New Delhi found that samples from AES patients like CSF, serum, urine, nasal swabs, throat swabs, rectal swabs, postmortem brain and liver biopsy were negative for JEV, Nipah virus, WNV and Chandipura virus [3]. The present study focuses on cases from Patna, which drains cases from all over Bihar. An infectious etiology could be determined in one third of the total cases studied, which comprised of scrub typhus, JEV, DV, WNV, ChikV in a good number of cases and *M*. *tuberculosis*, *S*. *pneumoniae*, *H*. *influenzae*, adenovirus, HSV-1, enterovirus, and measles virus occassionally.

For the first time, we detected scrub typhus in AES cases from Bihar, though *O*. *tsutsugamushi* is known to exist in this region [5]. *O*. *tsutsugamushi* has already been established to invade the central nervous system [8,9,16]. In fact in a recent prospective study from Laos, *O*. *tsutsugamushi* was detected in 12% patients with CNS infection and having evidence of bacterial or fungal infection [17]. Scrub typhus is easily treatable when diagnosed correctly, though untreated cases have a case fatality rate of 30–35% [5]. The differential diagnosis of scrub typhus is a long list, because of its nonspecific clinical and laboratory features, combined with limited diagnostic facilities in developing countries like India. Therefore, the clinicians need a high index of suspicion for detecting this neglected and treatable disease in cases with AES at least in endemic areas [8, 9, 12]. The clinicians may start specific treatment with doxycycline or azithromycin when scrub typhus is considered likely [5,16]. In the present study, we established the diagnosis of *O*. *tsutsugamushi* infection based upon an ELISA technique since IgM capture assays are the most sensitive tests for diagnosing recent rickettsial infections, as significant titers of IgM antibody appear by the end of first week [5,18]. Real time PCR could detect three extra cases, which we would have missed if we relied only on the serological test; samples from these cases were collected within the first week of illness thereby increasing the rickettsemia [5] detection. Studies recommend using whole blood/ buffy coat [19] for scrub typhus PCR and not serum/ CSF, which may account for its low positivity in the present study. We confirmed *O*. *tsutsugamushi* Gilliam-like strains presence, similar to those isolated from Vietnam and Thailand, by sequence analysis. In 2015, studies reported equal proportions of Karp-like and Kato-like strains from Northern India [20] and Gilliam-like strains from Vellore and Shillong but not from Northern India. Though, in 2007 studies reported Gilliam- like strains from Northern India [21]. This highlights the need for a comprehensive genotype study from this region, which will help in vaccine development as well as in understanding implications of strain variations and pathogenesis [22].

We tested the cases for *M*. *tuberculosis* since India is a country with a high burden of the disease and about 1% tuberculosis cases develop CNS complications. Moreover, the AES case definition given by the World Health Organization is very broad and includes viral encephalitis, bacterial meningitis, tubercular meningitis, cerebral malaria and acute disseminated encephalomyelitis [23]. Since CNS tuberculosis usually presents as acute to subacute meningitis with symptoms of less than two weeks duration, we can label these cases as having AES as per the clinical case definition. Identifying *M*. *tuberculosis* is important for initiating specific treatment in these patients, who would otherwise have higher chances of mortality and poor outcome.

Some cases showed co-positivity between the arboviruses. In a hyperendemic region, if a case is positive for more than one arbovirus antibodies, three possibilities exist a. cross-reactivity, the arboviruses share antigenic epitopes in the major envelope (E) protein due to which cross-reacting antibodies are produced [24]; b. pre-existing immunity due to previous flavivirus infection or vaccination [25]; c. co-infection, already reported in areas with high transmission rates varying from 2% in Gabon to 34% in Nigeria[26,27]. The limitation of the present study is that we could not accurately determine the etiological agent/ agents in these cases as we could not perform the gold standard plaque reduction and neutralization test (PRNT) due to logistic reasons. Similarly, cross-reactivity or co-infection in hyperendemic regions [28] may explain the co-positivity of scrub typhus and viruses, as was also indicated by the PCR results.

Obtaining both CSF and serum from an ailing child is not always possible. At times CSF tap cannot be performed owing to the low general condition of the patient or the amount of CSF tapped is low and just enough to do the cell counts and biochemistry for immediate patient management. At times venepuncture becomes difficult or in a few cases, the amount of blood obtained becomes a limiting factor. In the present study in about fifty percent cases, the samples obtained were either CSF or serum, hence all the tests could not be performed in each case.

The highlights of this pilot AES study were the detection of an infectious etiology in one-third of the AES cases, multiple etiologies, and the emergence of *O*. *tsutsugamushi* infection as an important causative agent of AES in Bihar, India. We need more comprehensive studies to confirm the findings of this study.
